# Enhancement of the Antioxidant Capacity of Thyme and Chestnut Honey by Addition of Bee Products

**DOI:** 10.3390/foods11193118

**Published:** 2022-10-07

**Authors:** Vanesa Sánchez-Martín, Paloma Morales, Amelia V. González-Porto, Amaia Iriondo-DeHond, Marta B. López-Parra, María Dolores Del Castillo, Xavier F. Hospital, Manuela Fernández, Eva Hierro, Ana I. Haza

**Affiliations:** 1 Departamento de Nutrición y Ciencia de los Alimentos, Sección Departamental de Nutrición y Ciencia de los Alimentos, Facultad de Veterinaria, Universidad Complutense, 28040 Madrid, Spain; 2 Laboratorio de Miel y otros Productos de la Colmena, Centro de Investigación Apícola y Agroambiental (CIAPA)-Instituto Regional de Investigación y Desarrollo Agroalimentario y Forestal de Castilla-La Mancha (IRIAF), Junta de Comunidades de Castilla La Mancha, 19180 Guadalajara, Spain; 3 Instituto de Investigación en Ciencias de la Alimentación, Consejo Superior de Investigaciones Científicas (CSIC)-Universidad Autónoma de Madrid (UAM), 28049 Madrid, Spain; 4 Departamento de Nutrición y Ciencia de los Alimentos, Sección Departamental de Farmacia Galénica y Tecnología Alimentaria, Facultad de Veterinaria, Universidad Complutense, 28040 Madrid, Spain

**Keywords:** antioxidant, honey, natural food preservative, natural food sweetener, phenolic compounds, propolis, royal jelly

## Abstract

Honey consumption and imports have increased in recent years, and it is considered by consumers to be a healthy alternative to more commonly used sweeteners. Honey contains a mixture of polyphenols and antioxidant compounds, and the botanical origin and geographical area of collection play an important role on its chemical composition. The present study investigated the physicochemical properties, total phenolic content and antioxidant capacity of Spanish thyme honey and chestnut honey, and their mixtures with royal jelly (2% and 10%) and propolis (2% and 10%). The analysis of the physicochemical parameters of both honey samples showed values within the established limits. Propolis showed the highest value of total phenolic content (17.21–266.83 mg GAE/100 g) and antioxidant capacity (DPPH, ORAC and ABTS assays; 0.63–24.10 µg eq. Tx/g, 1.61–40.82 µg eq. Tx/g and 1.89–68.54 µg eq. Tx/g, respectively), and significantly reduced ROS production in human hepatoma cells. In addition, mixtures of honey with 10% of propolis improved the results obtained with natural honey, increasing the value of total phenolic content and antioxidant capacity. A significant positive correlation was observed between total phenolic compounds and antioxidant capacity. Therefore, the antioxidant capacity could be attributed to the phenolic compounds present in the samples, at least partially. In conclusion, our results indicated that thyme and chestnut honey supplemented with propolis can be an excellent natural source of antioxidants and could be incorporated as a potential food ingredient with biological properties of technological interest, added as a preservative. Moreover, these mixtures could be used as natural sweeteners enriched in antioxidants and other bioactive compounds.

## 1. Introduction

The interest in exploring the benefits of bee products has grown in recent years [1,2,3,4]. Honey is a natural, sweet, and viscous product made by bees (*Apis mellifera*). It can be produced from nectar, plant secretions or excretions of different insects, which bees collect, transform and store for maturation [5]. Honey components include different sugars (glucose, fructose, maltose, and sucrose), minerals, vitamins, enzymes, various phytochemicals, and water, and the botanical origin and geographical area of collection play an important role in honey’s chemical composition [6,7]. Numerous health-promoting properties are linked to the phytochemicals present in honey, including phenolic acids, flavonols, and alkaloids [8]. Several preventive and therapeutic properties have been associated with honey, such as anti-inflammatory, antitumor, antimicrobial, antioxidant, and wound healing properties [9,10,11,12,13]. In general, we can identify two types of honey: multifloral or monofloral, derived from a combination of several botanical species or from a single flower variety, respectively. The distinctive physicochemical features of monofloral honey constitute a commercial advantage increasing its market value [7]. Lamiaceae family species are pollinated by honeybees, and, within this family, genre *Thymus* L. produces attractive nectar to bees for its phenylalanine and sucrose content [14]. Thyme honey is produced from flowers of thyme (*Thymus* spp.) by bees, and its organoleptic properties give it a great value [15]. Additionally, in bee pollen, species of the Fagaceae family were also present [16]. Chestnut honey is produced from flowers of the chestnut tree (*Castanea sativa*) by honeybees, and it is consumed mainly as a nutritional food [17]. Moreover, honey is used as a natural sweetener with great nutritional value and consumers are paying more attention to the potential benefits of choosing honey over other sugars in their regular diet [18].

Propolis is a resinous substance, of varied colors, used by the bees to protect and keep their holes safe and clean. It can be accumulated from different plants and is also known as “bee glue” [19]. Natural propolis consists of wax, pollen, resin and vegetable balsam, essential and aromatic oils, and other substances, such as polyphenols, flavonoids, or diterpenoids, depending on the type and geographical regions, botanical source, season, and bee preferences [20,21]. In addition, propolis has been reported to exhibit several health-promoting properties such as antitumoral, anti-inflammatory, wound restoring, antibacterial and antioxidant effects [22,23,24,25,26].

Royal jelly is a white or cream-colored substance secreted from worker bees’ hypopharyngeal and mandibular glands [19]. Worker bees feed this substance to the young larvae that will become queen bee. The chemical composition of royal jelly is varied, including water, sugar, lipids, proteins, phenols and flavonoids, free amino acids, vitamins and minerals [27]. Anti-inflammatory, antioxidant, antiproliferative, hepato-renal protective and wound healing properties have been attributed to royal jelly [28,29,30,31,32].

Reactive oxygen species (ROS) are involved in oxidative cell damage and have been associated with various diseases, such as cardiovascular disorders, cancer, arthritis, or aging, while antioxidant compounds reduce free radical activity playing a principal role in health [33,34]. The potential health benefits of phytochemicals have received extensive attention in the recent literature, focusing especially on compounds with high antioxidant properties [5,8,35]. Phenolic compounds are important antioxidants in honey, and more than 150 compounds have been investigated [36]. Their content is correlated with the color of the honey; a dark color indicates a higher phenolic content and antioxidant capacity [37,38].

Artificial preservatives are chemicals added to foods during the manufacturing process, and some of the most popular are sorbate, sulfite, and nitrite [39]. These preservatives may cause health problems, such as hypersensitivity, allergy, neurological damage, and cancer, so natural preservatives are an alternative, as they are considered safe, show antimicrobial and antioxidant effects, and extend the shelf life of food [39,40,41]. The use of phenolic compounds as potential biopreservatives in food has been recently demonstrated [42,43].

In this study, we have evaluated the physiochemical properties of Spanish thyme and chestnut honey. Moreover, for the first time, we have investigated the effect of the addition of bee products on their health-promoting properties. This study provides evidence that mixtures of honey with propolis could be added as food preservatives and natural sweeteners.

## 2. Materials and Methods

### 2.1. Raw Materials

Thyme (TH) and chestnut (CH) honey samples were obtained directly from beekeepers in each region at the Hive Products Laboratory of the Beekeeping and Agro-Environmental Research Center (CIAPA) of Marchamalo, Guadalajara (Spain). The scientific and common name, type, and family that form the principal flora of honey samples, as well as the geographic region of honey samples, royal jelly (RJ) and propolis (PR), are shown in Table 1. Regarding the geographic region of honey samples, Zamora is located at 5°44′40″ O and 41°30′22″ N longitude and latitude, and Toledo is located at 4°44′48″ O and 40°06′26″ N longitude and latitude. Samples were obtained in January 2021, and the analyses were performed within 3 months of collection. Thyme and chestnut honey samples were mixed with royal jelly and propolis (2–10%) to obtain 16 samples analyzed in this work (Table 2).

Samples (250 g) were homogenized in a laboratory blender, LB 400 W (with glass window in the door, blade speed (min^−1^): 240, fixed speed: 8 strokes/s). Propolis tincture was used (dissolved in 70% organic ethanol). For sample preparation, samples 1–16 were weighed and diluted in sterile distilled water at a final concentration of 1 g per ml of sample, except for sample number 15 (RJ), which was prepared at 0.5 g per ml of sample. Then, samples were filtered (0.45 µm) and stored at −20 °C [14]. Artificial honey (AH) was prepared by diluting 4.05 g of fructose, 3.35 g of glucose, 0.15 g of sucrose, and 0.75 g of maltose, in sterile distilled water (1 g/mL) (Table 2) [44].

### 2.2. Cell Culture

HepG2 cells were provided by Unidad de Terapias Farmacológicas and Unidad de Genética Molecular (Instituto de Investigación de Enfermedades Raras, Instituto de Salud Carlos III, Madrid), cultured in Dulbecco’s modified Eagle’s medium (Gibco, Gaithersburg, MD, USA), and supplemented with 10% *v/v* fetal bovine serum (heat-inactivated), 50 µg/mL streptomycin, 50 U/mL penicillin, and 50 µg/mL L-glutamine. Human hepatoma cells (HepG2) were incubated at 37 °C and 100% humidity in a 5% CO_2_ atmosphere [45].

### 2.3. Chemicals and Reagents

The following reagents were purchased from Sigma Chemical (Sigma-Aldrich, St. Louis, MO, USA): Folin-Ciocalteu reagent, gallic acid, 2,2-Diphenyl-1-picrylhydrazyl (DPPH), 6-hydroxy-2,5,7,8-tetramethyl-chroman-2-carboxylic acid (Trolox), 2,2′-azino-bis(3-ethylbenzothiazoline-6-sulfonic acid (ABTS), potassium persulfate (K_2_S_2_O_8_), sodium carbonate (Na_2_CO_3_), potassium phosphate monobasic (KH_2_PO_4_), sodium phosphate dibasic (Na_2_HPO_4_), fluorescein sodium, 2,2′-azobis(2-amidino-propane) dihydrochloride (AAPH), and N-Acetyl-L-cysteine (NAC). 2’,7’-dichlorodihydroflourescein diacetate (H_2_DCFDA) was obtained from Molecules Probes (Eugene, Oregon, USA). Sucrose, maltose, fructose, and glucose were purchased from Panreac Chemical, SLU (Barcelona, Spain). The water used was purified by the Milli-Q systems. Methanol used had a purity of 99.8%.

### 2.4. Analysis of the Physicochemical Properties of Thyme and Chestnut Honey Samples

Sample 1 (TH) and sample 6 (CH) were initially characterized by melissopalynological analysis to determine the botanical source. Pollen analysis was performed according to the methods established by the International Commission of Bee Botany [46,47].

Physicochemical properties of thyme and chestnut honey were analyzed at the Hive Products Laboratory of the Beekeeping and Agro-Environmental Research Center (CIAPA) of Marchamalo, Guadalajara (Spain). The pH was obtained by potentiometry at 20 °C in a 10% (*w/v*) solution of honey in boiled, distilled water (pH meter XS PC510 pH-meter, Eutech Instruments Pte Ltd, Singapore). Free acidity was determined by plotting the neutralization curve titrated with sodium hydroxide and calculating the pH of the equivalence point. Moisture was measured by refractometry at 20 °C (Abbé analog refractometer, Auxilab S.L., Navarra, Spain). Electrical conductivity was obtained at 20 °C in a 20% (*w/v*) solution of honey (dry matter basis) in deionized water employing a CDM-83 conductometer (Radiometer, Copenhagen, Denmark). Water activity (Aw) was measured using AquaLab Lite (Decagon Devices, Inc., USA).

Hydroxymethylfurfural (HMF) content was determined by reading UV absorbance at 284 nm (method approved by the International Honey Commission, 2009) with a Hitachi model U-1100 spectrophotometer [48]. Diastase activity (α-amylase), after the Schade method was quantified in Schade units per gram of honey [48]. One Schade unit is the total of enzymes that will transform 0.01 g of starch to the end-point in one hour at 40 °C under test conditions [49].

#### 2.4.1. Sugar Profile

Sugar content (glucose, fructose, and sucrose) was determined using Analyzer Y15 [50]. The Y15 is an automatic analyzer whose performance is based on an enzymatic method (enzymes included in the kit: hexokinase, phosphoglucose isomerase, glucose 6-phosphate dehydrogenase). This analyzer is a photometer with a thermostatizable cuvette at 37 °C. With this method, glucose content and the total glucose and fructose content from the sediment are determined. By enzymatic reaction, NADPH is generated, which will be measured by spectrometry at 340 nm. The content of both glucose and the sum of glucose and fructose is given in g/100 g. Fructose content is calculated from the difference between the content of the sum of glucose and fructose and the glucose content.

#### 2.4.2. Color Determination

The evaluation of color intensity was based on optical comparison using simple color grading as defined by Pfund and Lovibond [51,52]. In general, honey is marked according to the Pfund color scale, so Lovibond graders on a Pfund scale are used. Other more objective methods have also been used, such as the determination of all color parameters by the CIELAB L*a*b* three-dimensional method [52,53,54]. The CIELAB system is a reflection method (measuring geometry d80, illuminant D65, range 400–700 nm, observer 10°) performed on a Hitachi model U-1100 spectrophotometer (L* lightness, a* chromaticity +red/−green, b* chromaticity +yellow/−blue, C*ab chroma, hab, 10).

#### 2.4.3. Determination of Fat and Protein

Fat was analyzed using Soxhlet extraction. In this traditional method, samples are dried and ground, and stand in a thimble. The flask is heated, the solvent is evaporated and moved to the condenser, and the liquid is collected in the extraction chamber. This chamber contains the sample and, finally, the solvent passes through the sample and the fats go to the flask [55].

The Kjeldahl method was used to indirectly quantify protein concentration, from the nitrogen content of the sample [56]. This method involves three steps: digestion, distillation, and titration. Natural honey samples were digested by heating in the presence of sulfuric acid and catalysts, and organic nitrogen was converted to ammonium sulphate. Then, sodium hydroxide was added, and ammonium sulphate was converted to ammonia. Ammonia and an excess of boric acid formed ammonium borate. Finally, titration with acid determined the amount of borate [57].

### 2.5. Total Phenolic Compounds (TPC)

Folin-Ciocalteu adapted was used for the analysis of TPC in samples 1–17 [58]. Ten μL of sample or standard and 150 μL of Folin solution were added to a 96-well plate and incubated for 3 min at room temperature. Then, 50 µL of sodium carbonate solution were added. Samples were incubated for 2 h and the absorbance was read at 735 nm using a UV-Visible Spectrophotometer (BioTek Instruments, Winooski, VT, USA). Gallic acid (0.00–0.5 mg/mL) was used as standard. Measurements were performed in triplicate and results were expressed as mg GAE eq. per 100 g of sample (mg GAE/100 g of sample).

### 2.6. Antioxidant Capacity

#### 2.6.1. Determination of Radical Scavenging Capacity against DPPH

The antiradical activity of samples was determined by the 2,2,diphenyl-1-picrylhydrazyl (DPPH) test. In the presence of an antioxidant, the DPPH radical can accept an electron or hydrogen radical to become a stable molecule without dimerization [59]. In the presence of an antioxidant, the violet color of DPPH decays, and changes absorbance, which can be measured spectrophotometrically at 517 nm. Consequently, the higher the antioxidant capacity, the stronger the decolorization of the reagent [60].

Briefly, a stock solution of DPPH (0.125 g/L) in methanol was prepared. From the stock solution, the daily DPPH solution was prepared at a final concentration of 0.05 g/L and adjusted to an absorbance of 0.07 ± 0.02 at 517 nm. Each well of a 96-well microplate contained 273 µL of DPPH and 7 µL of samples 1–17 (final volume: 280 µL of assay solution). Trolox (0.00–1.2 mmol/L) was used as a standard. After incubation for 60 min in the dark, the absorbance of the reaction mixture was measured at 517 nm using a UV-Vis spectrophotometer. Measurements were performed in triplicate and results were expressed as µmol Trolox eq. per gram of sample (μmol TE/g of sample).

#### 2.6.2. Determination of the Oxygen Radical Absorbance Capacity (ORAC)

The Oxygen Radical Absorbance Capacity (ORAC) method estimates the ability of antioxidants to protect the fluorescent probe (fluorescein) from damage by free radicals. The protective effect of antioxidants is determined by calculating the area under the resulting kinetic curve (AUC) and subtracting this result from the AUC value of the blank reaction (without antioxidant compound). The resulting difference is the antioxidant protection conferred by the sample [61,62,63]. All reagents were prepared in 75 mM phosphate buffer (pH 7.4) and Trolox (5–95 µmol/L) was used as a standard. Each well of a 96-well microplate contained 150 µL of fluorescein (0.14 µM) and 25 µL of samples 1–17 (final volume: 200 µL of assay solution). After incubation for 30 min at 37 °C, 25 µL of AAPH (0.15 M) were added and fluorescence was measured every minute for 60 min at 37 °C, with emission and excitation wavelengths of 528 ± 20 nm and 485 ± 20 nm, respectively, using a microplate fluorescence reader. Measurements were performed in triplicate and results were expressed as µmol Trolox eq. per gram of sample (μmol TE/g of sample).

#### 2.6.3. ABTS Scavenging Assay

Cationic free radical scavenging capacity was evaluated, in samples 1–17, using the method of radical ABTS bleaching described by Re et al. and rectified by Oki et al. for use in a microplate [64,65]. ABTS stock solution was prepared by incorporating the ABTS radical into a potassium persulfate solution and allowing it to stand for 16 h at room temperature. The stock solution was diluted at 1:75 (*v/v*) in 5 mM of sodium phosphate buffer (pH 7.4) and adjusted to an absorbance of 0.7 ± 0.02 at 734 nm. Aqueous solutions of Trolox (0.0–200 μmol/L) were used for calibration. Absorbance was read using a UV–Visible Spectrophotometer (BioTek Instruments, Winooski, VT, USA). All measurements were performed in triplicate, and results were expressed as μmol Trolox eq. per gram of sample (μmol TE/g of sample).

### 2.7. Reactive Oxygen Species (ROS) Assay

ROS production was determined using 2′,7′-dichlorodihydrofluorescein diacetate (H_2_DCFDA). H_2_DCFDA crosses the cell membrane and is hydrolyzed by esterases to non-fluorescent dichlorofluorescein (DCFH). If ROS are present, this compound is oxidized to highly fluorescent dichlorofluorescein (DCF). HepG2 cells were cultured in Dulbecco’s Modified Eagle’s Medium, without phenol red and fetal bovine serum, and afterwards were treated with 25 µL of the corresponding sample (honey, royal jelly, propolis, and their mixtures, samples 1–17) or NAC (20 mM) for different time intervals (0.25–24 h). Then, 2 × 10^5^ cells were washed with PBS, loaded for 30 min with H_2_DCFDA (10 µM) and incubated at 37 °C. Cells were kept on ice and fluorescence intensity was measured immediately with FACSCalibur flow cytometer (Becton & Dickinson) and the CellQuest software. For each experiment, 10^4^ cells were analyzed and DCF fluorescence was expressed as a percentage of control.

### 2.8. Statistical Analysis

Data are expressed as the mean of three independent experiments ± SD. One-way analysis of variance (ANOVA) was performed. Statistical comparisons of the different treatments were performed using Tukey’s test at a 95% confidence interval, with a significance level of (α = 0.05). Values of *p* < 0.05 were considered statistically significant. Data were analyzed using the Statgraphics program.

## 3. Results and Discussion

### 3.1. Physicochemical Properties of Thyme and Chestnut Honey

A honey sample can be classified as monofloral honey when more than 45% of the pollen grains are from a single plant species; however, for thyme honey, it must be at least 18% [35]. Analyses carried out in the present study showed the monofloral origin of both honey samples, sample 1 (TH) and sample 6 (CH) (*Thymus*, 47.19%, and *Castanea sativa* Mill., 64.98%).

The study of the physicochemical properties of thyme honey (TH) and chestnut honey (CH) is based on the Spanish Royal Decree-Law (RD) 1049/2003 (Table 3) which approves the honey quality standard [66]. The pH value obtained for the thyme honey was 4.90 ± 0.01, and 5.31 ± 0.02 for the chestnut honey. Depending on the organic acids found in the honey, pH varies in a range between 3.5 and 5.5, since a value higher than 7 could promote the growth of microorganisms [61]. The pH of studied honey samples is between 3.5 and 5.5. Free acidity of the two honey samples, with values of 28.50 ± 0.43 meq/kg and 26.25 ± 0.35 meq/kg, was well below the limit of 50 meq/kg of honey established by the standard. Lafraxo et al. reported a value of 28.56 ± 1.54 meq/Kg for free acidity of thyme honey [67]. Dag et al. showed values between 18 and 25 meq/Kg for free acidity of chestnut honey [68]. Thyme and chestnut honey had pH and free acidity values within the limits accepted by quality standards, confirming the freshness of the honey.

Moisture content, which reflects the stability of honey against possible fermentation, must be less than 20%, as stated in this standard. Our data were 16.65 ± 0.50% for thyme honey and 15.83 ± 0.44% for chestnut honey. Our findings showed similarity with the results of Anjos et al. for Portuguese and Spanish honey samples [69]. Electrical conductivity is closely related to the mineral content, and the geographical and botanical origin of honey. Electrical conductivity value was 0.64 ± 0.10 mS/cm and 1.16 ± 0.05 mS/cm for thyme and chestnut honey, respectively. According to the standard, the values must be less than 0.8 mS/cm for thyme honey and more than 0.8 mS/cm for chestnut honey. Water activity in flower honey is in the range of 0.479 to 0.557, and water activity values were 0.53 ± 0.01 and 0.51 ± 0.01 for thyme honey and chestnut honey, respectively [70].

Both honey samples showed no HMF and a high diastase index, which leads to deduce that they are honey samples of recent production, in optimal conditions. Fallico et al. reported no HMF in chestnut honey, and Rodríguez et al. described values between 0.9 mg/kg and 10 mg/kg for thyme honey (lower HMF data correspond to higher pollen grain content) [71,72]. Regarding diastase activity, several studies have shown values from 20.2 to 50.6 for thyme honey, and from 19.10 to 24.10 or from 17.45 to 19.20 for chestnut honey [72,73,74]. Our data are clearly far from the limits for these parameters (not exceeding 40 mg/kg and being higher than 8 for HMF and diastase index, respectively).

Honey is mainly composed of sugars and the overall content of glucose and fructose should not be less than 60 g/100 g of honey for flower honey. For thyme honey, this content was 63.80 g/100 g, and for chestnut honey, 67.37 g/100 g. This also qualifies the chestnut honey as flower honey and not honeydew honey, which would mean a lower sugar content. Percentages of glucose and fructose are slightly lower than those obtained in the study of León-Ruiz et al., but within the range of the results of Xagoraris et al. and Escuredo et al. [75,76,77]. Both honey samples showed a sucrose content lower than 5 g/100 g, with values of 1.25 ± 0.08 g/100 g for thyme honey and 0.68 ± 0.01 g/100 g for chestnut honey.

Color is an additional parameter that is not included in the standard. However, its analysis provides useful information on botanical origin (Table 3). Light amber color (Pfund range between 51 and 85, X10 between 0.4451 and 0.5255) for thyme honey and an amber to dark amber color (Pfund range of values greater than 114, X10 between 0.5279 and 0.5957) for chestnut honey were obtained. Results of León-Ruiz et al. showed a higher clarity value for thyme honey compared to chestnut honey, which is in accordance with our data [75].

Results obtained after the analysis of the different parameters showed the freshness and quality conditions of thyme and chestnut honey samples, according to the Spanish Royal Decree-Law (RD) 1049/2003 [66].

Fat, as well as sugar, plays a critical role in energy metabolism, and protein is also considered a macronutrient [78]. Fat and protein contents were measured in thyme and chestnut honey (Table 3). Results indicated that both honey samples had a fat content of less than 0.50%, similar to the value obtained by Mustafa et al. (0.54%) for natural honey [79]. Focusing on protein, chestnut honey had a higher protein value (0.61 ± 0.01%) than thyme honey (0.45 ± 0.01%). Some studies have also shown a higher protein content in chestnut honey compared to thyme honey [80,81].

### 3.2. Total Phenolic Compounds (TPC) of Spanish Thyme and Chestnut Honey and Their Mixtures with Royal Jelly and Propolis

Data on the TPC in samples 1–17 are summarized in Table 4. The TPC of natural honey, honeybee products, and their mixtures varied between 17.21–266.83 mg GAE/100 g. Results from TPC determinations in samples showed that sample 16 (PR) (266.83 ± 19.80 mg GAE/100 g of sample) presented significantly the highest (*p* < 0.05) value of TPC, compared to sample 15 (RJ) (17.21 ± 0.71 mg GAE/100 g) which presented the lowest value. Honey evaluated by other authors also presented values of TPC in the range obtained in the present study [82].

Thyme and chestnut honey, samples 1 (TH) and 6 (CH), did not present significant differences (*p* > 0.05) in the total content of phenolic compounds, in line with the data reported by León-Ruiz et al. [83]. Among the mixtures of honey and honeybee products, sample 5 (TH + 10PR) had the highest value of TPC (107.42 ± 9.18 mg GAE/100 g), followed by sample 12 (TH + 10RJ + 10PR) (83.21 ± 3.95 mg GAE/100 g), sample 10 (CH + 10PR) (72.83 ± 44.71 mg GAE/100 g) and sample 14 (CH + 10RJ + 10PR) (66.23 ± 2.01 mg GAE/100 g). All the samples supplemented with 10% of propolis showed the highest value of TPC. In general, mixtures of thyme and chestnut honey with royal jelly (sample 2, TH + 2RJ, sample 3, TH + 10RJ, sample 7, CH + 2RJ, and sample 8, CH + 10RJ) and mixtures of thyme and chestnut honey with 2% of royal jelly and 2% of propolis (sample 11 and sample 13) showed similar values to samples 1 (TH) and 6 (CH). The TPC value of sample 4 (TH + 2PR) was higher than that of sample 9 (CH + 2PR). Finally, sample 15 (RJ) obtained the lowest value of all the samples analyzed (17.21 ± 0.71 mg GAE/100 g). Artificial honey (sample 17, AH) obtained the lowest values of TPC. These values agreed with those previously described [84].

### 3.3. Antioxidant Capacity of Spanish Thyme and Chestnut Honey and Their Mixtures with Royal Jelly and Propolis

Results from the in vitro antioxidant capacity determinations in samples 1–17 (Table 4), showed that sample 16 (PR) (24.10 ± 1.17 µmol eq. Tx/g, 40.82 ± 0.56 µmol eq. Tx/g, 68.54 ± 3.91 µmol eq. Tx/g) had the highest antioxidant capacity compared to sample 15 (RJ) (0.63 ± 0.57 µmol eq. Tx/g, 1.89 ± 0.35 µmol eq. Tx/g, 1.61 ± 0.78 µmol eq. Tx/g) and samples 1 (TH) (1.37 ± 0.10 µmol eq. Tx/g, 4.17 ± 0.99 µmol eq. Tx/g, 7.36 ± 0.55 µmol eq. Tx/g) and 6 (CH) (1.09 ± 0.12 µmol eq. Tx/g, 3.59 ± 0.87 µmol eq. Tx/g, 4.95 ± 0.30 µmol eq. Tx/g) by the three methods evaluated, DPPH, ORAC, and ABTS assays, respectively. Our results are in agreement with previous studies reporting the highest antioxidant power of propolis compared to other bee products [85]. The chemical composition of propolis is heterogeneous depending on the origin of the sample, which is clearly related to the geographic area, the flora surrounding the hive, climatic characteristics, and honeybee species [86]. Propolis samples from a certain area have similar antioxidant capacity. Furthermore, antioxidant capacity of propolis depended on the month of production [87]. In other words, the possible health-related effects due to the antioxidant properties of propolis may well depend on its origin [88]. The antioxidant potential of royal jelly differs depending on the age of the larvae and the time of harvest after the larval transfer to the beehives. The royal jelly harvested from the 1 day old larvae showed higher antioxidant power [89]. In addition, higher content in phenolic compounds was determined in the royal jelly harvested 24 h after the graft compared to that of 48 or 72 h [89].

Sample 1 (TH) showed significantly higher values (*p* < 0.05) only in the ABTS assay (7.36 ± 0.55 µmol eq. Tx/g honey) compared to sample 6 (CH) (4.95 ± 0.30 µmol eq. Tx/g honey). Previous studies differed in the results of antioxidant capacity for thyme and chestnut honey. In line with our results, Sipahi et al. and León-Ruiz et al. determined that thyme honey showed a higher antioxidant capacity than chestnut honey [83,90]. In addition, León-Ruiz et al., in their studies of different kinds of honey from Spain, reported that thyme honey had a higher content of vitamin C, which could be responsible, in part, for its greater antioxidant capacity compared to chestnut honey, whose vitamin C values were lower [83]. However, other studies have reported that the antioxidant capacity of chestnut honey is higher than that quantified for thyme honey [91]. In line with this, Beretta et al. reported that honey samples with a dark color (such as chestnut honey) have a higher antioxidant capacity than lighter colored honey samples (such as thyme honey) [92]. This fact highlights the relevance of the geographical and botanical origin [83].

In general, the addition of 10% propolis increased the antioxidant capacity of samples in the following order: 5 (TH + 10PR), 12 (TH + 10RJ + 10PR), 10 (CH + 10PR), and 14 (CH + 10RJ + 10PR). Our results are in agreement with Juszczak et al. [93]. Their studies indicated that the addition of small quantities of propolis, even at levels below 1%, results in a significant increase (12.96 ± 3.62% for honey and 34.08 ± 9.73% for honey with propolis in DPPH assay; 13.63 ± 4.51% for honey and 53.54 ± 20.91% for honey with propolis in ABTS assay) in the antioxidant capacity of honey. Artificial honey (sample 17, AH) obtained the lowest values of overall antioxidant capacity. These values are confirmed by the literature data [94,95].

In vitro antioxidant capacity should not be determined by a single type of antioxidant assay, mainly due to the complexity of the food matrix, the multiple chemical diversity of antioxidant substances, and the variety of free radical reaction mechanisms related to oxidative processes. In our study, results of the DPPH, ORAC, and ABTS assays differ from each other in the values obtained, nevertheless, the same tendency of antioxidant capacity in honey and bee product samples has been observed, which is probably related to the different mechanisms of action of each assay. ABTS assay is based on the spectrophotometric measurement of the neutralization of specific cation radicals, DPPH assay is a rapid and sensitive colorimetric reaction based on the neutralization of a nitrogen radical, while in ORAC assay, the oxygen radical absorption ability is estimated kinetically with fluorometric detection. The antioxidant capacity in the free radical scavenging process is most commonly assessed by ABTS (allows to measure the activity of compounds of a hydrophilic and lipophilic nature) and/or DPPH (only allows to dissolve in organic medium) methods whereas the ORAC method allows evaluating the capacity of an antioxidant to preserve the target molecule against its oxidative process [96,97,98,99].

Data on total phenolic compounds and overall antioxidant capacity measured by DPPH, ORAC, and ABTS of honey samples, honeybee products, and their mixtures showed a positive linear correlation (Figures S1–S3, see Supplementary Material). Correlations coefficients of 0.99, 0.97, and 0.99 have been obtained for DPPH, ORAC, and ABTS assays, respectively. The correlation obtained suggests the contribution of phenolic compounds to the overall antioxidant properties of samples. Several studies associated the antioxidant capacity of honey and honeybee products mainly with the content of phenolic compounds [60,100,101,102,103]. In this study, samples with the highest total phenolic content exhibited the highest antioxidant capacity. Moreover, polyphenols enhance the antioxidant properties of proteins. Jiang et al. demonstrated that phosvitin combined with gallic acid improves the antioxidant ability of this protein [104]. In other studies, it has been shown the higher antioxidant capacity of the conjugate of isolated soy protein and tea polyphenol [105]. The interactions between proteins and polyphenols are also involved in protein functionality and digestibility [106].

### 3.4. Time Course of ROS Production induced by Spanish Thyme and Chestnut Honey and Their Mixtures with Royal Jelly and Propolis

**Figure 1:**
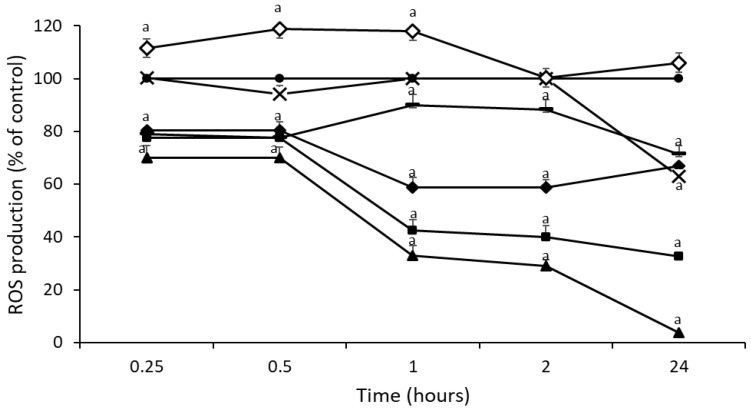
Time course of ROS production in untreated HepG2 cells (●) and treated with NAC (■), sample 1 (TH) (◆), sample 6 (CH) (▬), sample 15 (RJ) (X), sample 16 (PR) (▲), and sample 17 (AH) (◇). Significant difference from control ^a^ *p* < 0.05.

The study of the antioxidant capacity by DPPH, ORAC, and ABTS assays has been completed with the evaluation of ROS production in HepG2 cells. DCF fluorescence was measured by flow cytometry and expressed as a percentage of the control. N-acetyl-L-cysteine (NAC) was used as an antioxidant standard. As observed in Figure 1, samples 1 (TH), 6 (CH), 15 (RJ), and 16 (PR) significantly reduced ROS production (*p* < 0.05) compared to untreated cells. However, sample 17 (AH) did not have this effect. In agreement with our results, it has been reported that buckwheat, heather, lavender, rosemary, and oak honey reduce ROS levels in various cell lines [44,107]. Moreover, royal jelly produces a reduction of intracellular ROS in lipopolysaccharide (LPS)- treated macrophages, and propolis reduces ROS production in macrophage and human keratinocyte cell lines [108,109,110].

Samples 1 (TH), 6 (CH), and 16 (PR) reduced ROS levels over time, while sample 15 (RJ) was only effective at 24 h. Of all samples (Figure 1), only sample 16 (PR) was able to decrease ROS production in a similar manner as the antioxidant standard used, improving the data obtained with NAC at all times studied. Propolis showed a reduction of more than 50% at 1 and 2 h, and at 24 h the percentage of reduction was 96% compared to 68% for NAC. These results are in line with those on total phenolic content and overall antioxidant activity measured by DPPH, ORAC, and ABTS assays reported in the present work. Sample 16 (PR) was the sample with the highest total phenolic content and antioxidant capacity.

**Figure 2:**
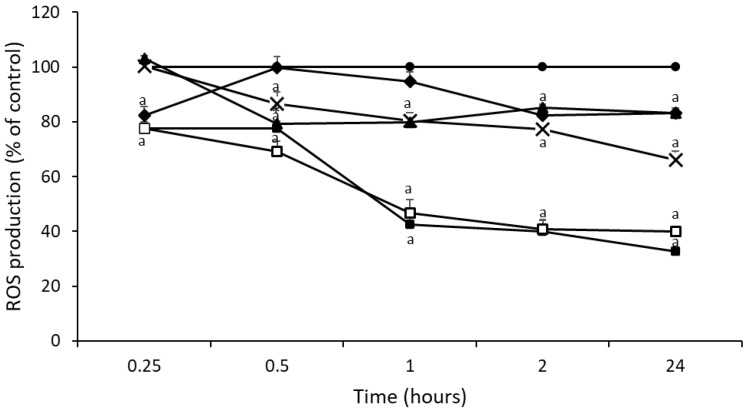
Time course of ROS production in untreated HepG2 cells (●) and treated with NAC (■), sample 2 (TH + 2RJ) (▲), sample 3 (TH + 10RJ) (◆), sample 4 (TH + 2PR) (X), and sample 5 (TH + 10PR) (□). Significant difference from control ^a^ *p* < 0.05.

The time course of ROS production induced by thyme honey mixtures with royal jelly or propolis in HepG2 cells is shown in Figure 2. Sample 5 (TH + 10PR) showed the greatest reduction (*p* < 0.05) of ROS levels (53% at 1 h and 59% at 2 h and 24 h), with similar values to NAC. Moreover, sample 5 (TH + 10PR) presented a greater reduction in ROS levels than thyme honey (Figure 1). The addition of propolis to thyme honey (sample 4, TH + 2PR, and sample 5, TH + 10PR) caused a higher reduction of ROS levels than when royal jelly was added (sample 2, TH + 2RJ, and sample 3, TH + 10RJ). According to our findings, sample 5 (TH + 10PR) had a high TPC value and antioxidant capacity (DPPH, ORAC and ABTS assays) among all the thyme honey samples under study.

**Figure 3:**
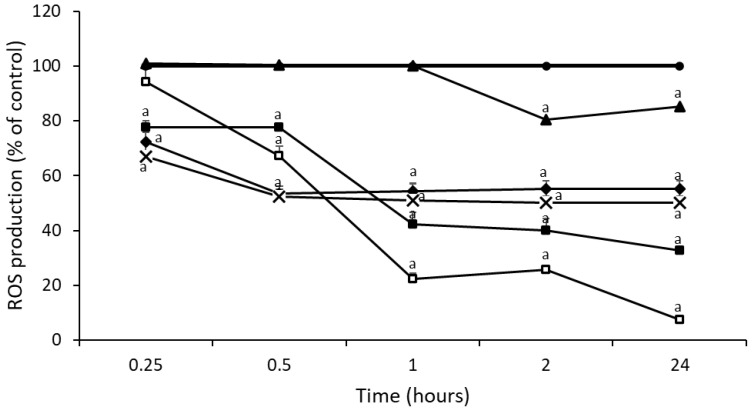
Time course of ROS production in untreated HepG2 cells (●) and treated with NAC (■), sample 7 (CH + 2RJ) (▲), sample 8 (CH + 10RJ) (◆), sample 9 (CH + 2PR) (X), and sample 10 (CH + 10PR) (□). Significant difference from control ^a^ *p* < 0.05.

Figure 3 shows the effect of chestnut honey mixtures with royal jelly or propolis on ROS production in HepG2 cells. ROS levels decreased with all mixtures, however, mixtures of chestnut honey and propolis (sample 9, CH + 2PR, and sample 10, CH + 10PR) showed the highest reduction of ROS levels compared to mixtures with royal jelly (sample 7, CH + 2RJ, and sample 8, CH + 10RJ). Sample 10 (CH + 10PR) showed the greatest reduction (78%, 75%, and 93% at 1, 2, and 24 h, respectively) and better results than NAC. Based on our previous results of antioxidant capacity by DPPH, ORAC and ABTS assays and total phenolic content, sample 10 (CH + 10PR) had the highest value of TPC and a high antioxidant capacity of the chestnut honey mixtures. Moreover, samples 8 (CH + 10RJ), 9 (CH + 2PR) and 10 (CH + 10PR) showed a greater reduction in ROS levels compared to chestnut honey.

**Figure 4:**
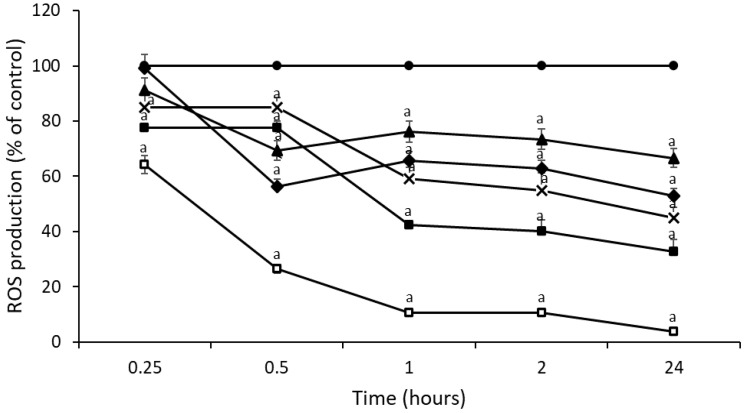
Time course of ROS production in untreated HepG2 cells (●) and treated with NAC (■), sample 11 (TH + 2RJ + 2PR) (▲), sample 12 (TH + 10RJ + 10PR) (◆), sample 13 (CH + 2RJ + 2PR) (X), and sample 14 (CH + 10RJ + 10PR) (□). Significant difference from control ^a^ *p* < 0.05.

Natural honey samples supplemented with both royal jelly and propolis (samples 11–14) also reduced ROS production in HepG2 cells compared to control cells (Figure 4). A significant reduction in ROS production (*p* < 0.05) after treatment with sample 14 (CH + 10RJ + 10PR) (74% at 0.5 h, 90% at 1, and 2 h, 96% at 24 h) was found. In addition, this sample improved the results obtained for NAC at all times studied.

In summary, 16 of 17 samples studied in the present work reduced intracellular ROS production in HepG2 cells compared to untreated cells. Samples 14 (CH + 10RJ + 10PR) and 16 (PR) showed the best results, followed by sample 10 (CH + 10PR) and sample 5 (TH + 10PR). Supplementation of thyme and chestnut honey with 10% propolis had a greater effect on the decrease of ROS levels in HepG2 cells.

Taking into account all of our results, samples with the highest phenolic content and overall antioxidant capacity (DPPH, ORAC, and ABTS) were in this order: samples 16 (PR), 5 (TH + 10PR), 10 (CH + 10PR), 12 (TH + 10RJ + 10PR), and 14 (CH + 10RJ + 10PR). Samples 16 (PR), 14 (CH + 10RJ + 10PR), 10 (CH + 10PR), and 5 (TH + 10PR) reduced intracellular ROS formation in HepG2 cells. Propolis showed the highest values of total phenolic content and antioxidant capacity among all the studied samples, and its addition at 10% on thyme and chestnut honey significantly enhanced (*p* < 0.05) their total phenolic content and antioxidant capacity. In line with our results, Braik et al. demonstrated that propolis, with a high antioxidant capacity by DPPH assay, reduces ROS production in isolated cardiac mitochondria and protects the heart from oxidative stress damage [111]. In the study of Mendez-Pfeiffer et al., treatment of B-cell lymphoma cells with propolis (25 and 50 µg/mL) reduced intracellular ROS formation and showed high antioxidant capacity by DPPH assay (propolis reduced DPPH radical up to 80% at 100 µg/mL) [112]. Szliszka et al. also reported the strong antioxidant power of propolis by employing ABTS (ED_50_ of 40.6 μg/mL), DPPH (ED_50_ of 24.1 μg/mL), and ROS formation on phorbol myristate acetate (PMA)-activated macrophages (0.1–10 μg/mL) [113].

High sugar consumption has been related to an increased risk of obesity, type II diabetes, cardiovascular diseases, and cancer. Consumers are concerned about their sugar consumption and are looking for healthier food options, such as sugar from natural sources [114]. Thus, U.S. honey consumption and imports have increased in recent years, being a normal component of the diet, and it is considered by consumers as a healthy alternative to the most commonly used sweeteners [115]. In addition, while other natural sweeteners (stevia or agave nectar) require further processing, honey is a good option for consumers who choose products with little processing [116]. The consumption of honey stands out for its physicochemical and biological properties. Zheng et al. demonstrated that honey supplementation in mice diets decreased total serum cholesterol and triglycerides, and increased high-density lipoprotein cholesterol and antioxidant enzyme levels, compared to mixed sugars [117]. Chen et al. reported that honey suppressed insulin resistance via maintaining glucose and lipid homeostasis in mice [118]. In humans, honey supplementation reduced pain and symptoms of diabetic neuropathy after three months [119].

Furthermore, natural preservatives are getting more attention, and consumers have shown an increasing interest in products with natural antioxidants, which has encouraged food industries to research new natural food additives [120]. Moreover, the interest in phenolic compounds is growing in the food industry, related to their antioxidant capacity. For example, meat products are susceptible to lipid oxidation and the new trends are to use antioxidants from natural sources; in this way, bee pollen retarded lipid oxidation in pork sausage storage at 4 °C for 30 days [42]. The study of Bobade et al. has investigated the effect of honey on the techno-functional and bioactive properties of whole-grain corn flour cereals, and the results showed that honey addition lowered the loss of phenolic content during the extrusion process and improved the antioxidant capacity of breakfast cereals [121]. Mafra et al. demonstrated that red propolis hydroalcoholic extract protects tilapia salami against chemical and microbial deterioration and prevents putrefaction [122]. A green propolis ethanolic extract improved the antioxidant capacity in candies and has the potential for use as a functional ingredient in jelly candies [123].

Based on our results, samples supplemented with 10% of propolis show high values of total phenolic content and antioxidant capacity. Therefore, these results support that thyme and chestnut honey mixtures with 10% propolis could be incorporated as a potential food ingredient with biological properties of technological interest. Thus, samples 5 (TH + 10PR), 10 (CH + 10PR), 12 (TH + 10RJ + 10PR), 14 (CH + 10RJ + 10PR), and 16 (PR) could be used as natural preservatives for their antioxidant capacity in food to reduce or replace the synthetic preservatives, and to offer the consumer safe and good quality products with an added value. Moreover, these mixtures could be used as a natural sweetener enriched in antioxidants. According to the data obtained, honey mixtures with propolis could be considered a complex matrix with potential as a food supplement for the food industry, being a source of antioxidants and other bioactive compounds. In addition, our results could contribute to the promotion of bee products with a long tradition and available for all consumers.

## 4. Conclusions

The present work analyzed the effect of the addition of royal jelly and/or propolis on the total phenolic content and antioxidant capacity of thyme and chestnut honey. The addition of 10% propolis to honey significantly enhanced their total phenolic content and antioxidant power. Therefore, our data support the supplementation of thyme and chestnut honey with propolis as an excellent natural strategy for improving their chemical composition and health-promoting properties, and could be incorporated as a food ingredient with biological properties of technological interest, added as a preservative. In addition, these mixtures could be used as a natural sweetener enriched in antioxidants and other bioactive compounds.

## Figures and Tables

**Table 1:** Honey and bee products samples.

Honey and Bee Products	Scientific and Common Name	Family	Geographic Region	Type
Thyme honey	*Thymus* spp. Thyme	Lamiaceae	Spain (Zamora)	Monofloral
Chestnut honey	*Castanea sativa* Chestnut	Fagaceae	Spain (Toledo)	Monofloral
Royal jelly	-	-	France	-
Propolis tincture *	-	-	Spain (Zamora)	-

* Propolis extract dissolved in 70% organic ethanol.

**Table 2:** Honey and bee products samples and mixtures.

Sample	Description	Code
1	Thyme honey	TH
2	Thyme honey + 2% royal jelly	TH + 2RJ
3	Thyme honey + 10% royal jelly	TH + 10RJ
4	Thyme honey + 2% propolis	TH + 2PR
5	Thyme honey + 10% propolis	TH + 10PR
6	Chestnut honey	CH
7	Chestnut honey + 2% royal jelly	CH + 2RJ
8	Chestnut honey + 10% royal jelly	CH + 10RJ
9	Chestnut honey + 2% propolis	CH + 2PR
10	Chestnut honey + 10% propolis	CH + 10PR
11	Thyme honey + 2% royal jelly + 2% propolis	TH + 2RJ + 2PR
12	Thyme honey + 10% royal jelly + 10% propolis	TH + 10RJ + 10PR
13	Chestnut honey + 2% royal jelly + 2% propolis	CH + 2RJ + 2PR
14	Chestnut honey + 10% royal jelly + 10% propolis	CH + 10RJ + 10PR
15	Royal jelly	RJ
16	Propolis	PR
17	Artificial honey	AH

**Table 3:** Physicochemical properties of thyme and chestnut honey.

Parameter	Thyme Honey	Chestnut Honey
pH (u)	4.90 ± 0.01	5.31 ± 0.02
Free acidity (meq/kg)	28.50 ± 0.43	26.25 ± 0.35
Moisture (%)	16.65 ± 0.50	15.83 ± 0.44
Electrical conductivity (mS/cm)	0.64 ± 0.10	1.16 ± 0.05
Water activity (u)	0.53 ± 0.01	0.51 ± 0.01
Hydroxymethylfurfural (mg/kg)	0.00 ± 0.00	0.00 ± 0.00
Diastase (Schade units)	21.10 ± 0.42	30.00 ± 0.55
Glucose (g/100g)	26.40 ± 0.12	25.80 ± 0.18
Fructose (g/100g)	37.40 ± 0.26	41.57 ± 0.15
Glucose + Fructose (%)	63.80	67.37
Sucrose (g/100g)	1.25 ± 0.08	0.68 ± 0.01
X10 (u)	0.50 ± 0.01	0.55 ± 0.02
Y10 (u)	0.46 ± 0.01	0.44 ± 0.01
Z10 (u)	0.04 ± 0.01	0.01 ± 0.01
L*10 (u)	66.90 ± 0.48	44.90 ± 0.39
a*10 (u)	16.20 ± 0.02	26.24 ± 0.12
b*10 (u)	81.79 ± 0.11	72.75 ± 0.09
C*ab (u)	83.38 ± 0.17	77.34 ± 0.20
hab, 10 (u)	78.79 ± 0.10	70.16 ± 0.18
Pfund (u)	83.00 ± 0.58	118.00 ± 1.10
Turbidity (u)	0.12 ± 0.01	0.26 ± 0.01
Fat (%)	<0.50	<0.50
Protein (%)	0.45 ± 0.01	0.61 ± 0.01

Data are expressed as the means ± standard deviation (n = 3) (except for glucose + fructose and fat).

**Table 4:** TPC and antioxidant capacity of natural honey and supplemented with royal jelly and propolis.

Sample	Code	TPC (mg eq. GAE/100 g of Sample)	DPPH (µmol eq. Tx/g of Sample)	ORAC (µmol eq. Tx/g of Sample)	ABTS (µmol eq. Tx/g of Sample)
1	TH	38.68 ± 5.93 ^cd^	1.37 ± 0.10 ^c–e^	4.17 ± 0.99 ^c^	7.36 ± 0.55 ^de^
2	TH + 2RJ	39.38 ± 3.75 ^d^	1.51 ± 0.16 ^de^	4.33 ± 0.53 ^c^	7.94 ± 0.56 ^ef^
3	TH + 10RJ	38.32 ± 1.67 ^cd^	1.30 ± 0.14 ^b–e^	4.34 ± 1.15 ^c^	6.64 ± 0.48 ^b–e^
4	TH + 2PR	52.27 ± 4.76 ^e^	2.61 ± 0.17 ^fg^	13.95 ± 0.63 ^e^	9.77 ± 0.72 ^fg^
5	TH + 10PR	107.42 ± 9.18 ^h^	7.19 ± 0.24 ^j^	19.04 ± 1.44 ^f^	25.96 ± 1.05 ^i^
6	CH	35.41 ± 2.14 ^cd^	1.09 ± 0.12 ^b–d^	3.59 ± 0.87 ^bc^	4.95 ± 0.30 ^bc^
7	CH + 2RJ	31.32 ± 1.71 ^cd^	0.96 ± 0.08 ^b–d^	3.38 ± 0.39 ^bc^	5.04 ± 0.41 ^bc^
8	CH + 10RJ	26.05 ± 1.77 ^bc^	0.72 ± 0.07 ^a–c^	5.05 ± 0.69 ^c^	4.46 ± 0.30 ^b^
9	CH + 2PR	38.87 ± 3.68 ^d^	1.43 ± 0.08 ^c–e^	10.47 ± 0.70 ^d^	6.87 ± 0.81 ^c–e^
10	CH + 10PR	72.83 ± 4.71 ^fg^	3.44 ± 0.17 ^h^	12.92 ± 0.78 ^de^	11.99 ± 0.31 ^gh^
11	TH + 2RJ + 2PR	39.75 ± 2.50 ^de^	1.98 ± 0.08 ^ef^	4.62 ± 0.41 ^c^	7.43 ± 0.50 ^de^
12	TH + 10RJ + 10PR	83.21 ± 3.95 ^g^	4.37 ± 0.19 ^i^	14.74 ± 1.30 ^e^	12.73 ± 0.69 ^h^
13	CH + 2RJ + 2PR	35.06 ± 1.56 ^cd^	1.14 ± 0.14 ^b–d^	5.27 ± 1.03 ^c^	5.36 ± 0.16 ^b–d^
14	CH + 10RJ + 10PR	66.23 ± 2.01 ^f^	2.88 ± 0.18 ^gh^	14.11 ± 1.05 ^e^	11.34 ± 0.27 ^gh^
15	RJ	17.21 ± 0.71 ^b^	0.63 ± 0.57 ^ab^	1.61 ± 0.78 ^ab^	1.89 ± 0.35 ^a^
16	PR	266.83 ± 19.80 ^i^	24.10 ± 1.17 ^k^	40.82 ± 0.56 ^g^	68.54 ± 3.91 ^j^
17	AH	0.81 ± 0.32 ^a^	0.16 ± 0.02 ^a^	0.03 ± 0.01 ^a^	0.08 ± 0.01 ^a^

Data are expressed as the means ± standard deviation (n = 3). Values in each column with different letters differ significantly (Tukey test, *p* < 0.05). Tx: Trolox.

## Data Availability

All relevant data are within the manuscript and individual data could be accessed from corresponding author upon reasonable request.

## Supplementary Materials

The following supporting information can be downloaded at: https://www.mdpi.com/article/10.3390/foods11193118/s1, Figure S1: The fitted line plot linear regression model of total phenolic content (TPC) against DPPH assay; Figure S2: The fitted line plot linear regression model of total phenolic content (TPC) against ORAC assay; Figure S3: The fitted line plot linear regression model of total phenolic content (TPC) against ABTS assay.
