# Supplementary material for: Enhancement of the Antioxidant Capacity of Thyme and Chestnut Honey by Addition of Bee Products
Source: Foods. 2022 Oct 7;11(19):3118. doi: 10.3390/foods11193118 (PMC9564292; doi:10.3390/foods11193118)
Supplement: Supplementary file 1 [file foods-11-03118-s001.zip › foods-1935229-supplementary.pdf]

Enhancement of the antioxidant capacity of thyme and chestnut honey by addition of bee products

Vanesa Sánchez-Martín [1], Paloma Morales [1], Amelia V. González-Porto [2], Amaia Iriondo-DeHond [3], Marta B. López-Parra [3], María Dolores Del Castillo [3], Xavier F. Hospital [4], Manuela Fernández [4], Eva Hierro [4] and Ana I. Haza [1,*]

[1] Departamento de Nutrición y Ciencia de los Alimentos, Sección Departamental de Nutrición y Ciencia de los Alimentos, Facultad de Veterinaria, Universidad Complutense, 28040 Madrid, Spain

[2] Laboratorio de Miel y otros Productos de la Colmena, Centro de Investigación Apícola y Agroambiental (CIAPA)-Instituto Regional de Investigación y Desarrollo Agroalimentario y Forestal de Castilla-La Mancha (IRIAF), Junta de Comunidades de Castilla La Mancha, 19180 Guadalajara, Spain

[3] Instituto de Investigación en Ciencias de la Alimentación, Consejo Superior de Investigaciones Científicas (CSIC)-Universidad Autónoma de Madrid (UAM), 28049 Madrid, Spain

[4] Departamento de Nutrición y Ciencia de los Alimentos, Sección Departamental de Farmacia Galénica y Tecnología Alimentaria, Facultad de Veterinaria, Universidad Complutense, 28040 Madrid, Spain

* Correspondence: hanais@ucm.es; Tel.: +34-91-394-37-47

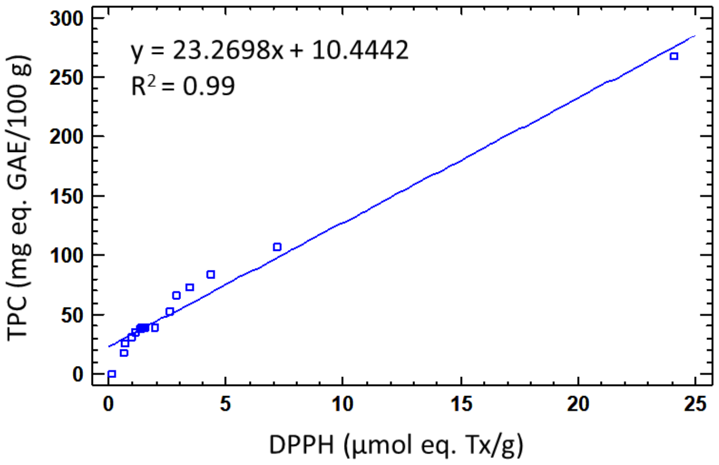

Figure S1. The fitted line plot linear regression model of total phenolic content (TPC) against DPPH assay.

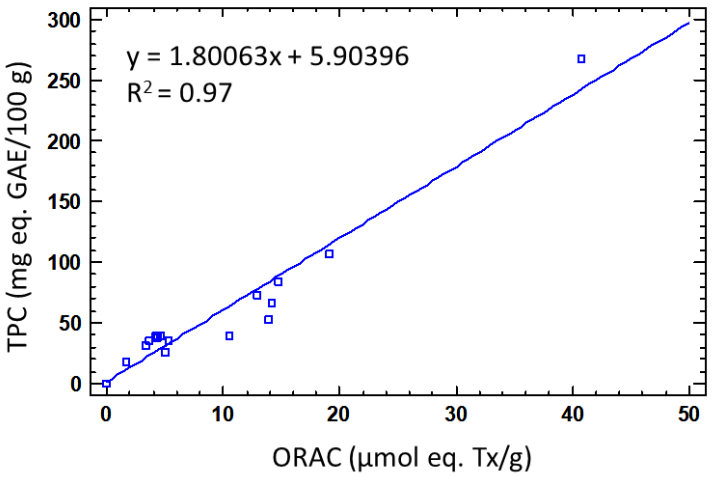

Figure S2. The fitted line plot linear regression model of total phenolic content (TPC) against ORAC assay.

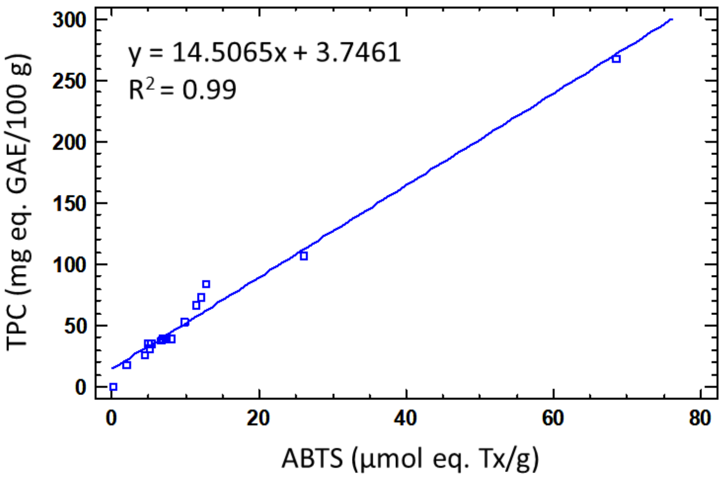

Figure S3. The fitted line plot linear regression model of total phenolic content (TPC) against ABTS assay.
